# Component-Level Prognostic Evaluation of Clinical, Inflammatory, and Renal-Protein Markers for 30-Day Mortality in Older Patients with Perforated Peptic Ulcer

**DOI:** 10.3390/jcm15187287

**Published:** 2026-09-19

**Authors:** Orçun Yalav, Serdar Gumus, Burak Aydoğan, Mustafa Alçın, İshak Aydın, Uğur Topal, Atılgan Tolga Akçam

**Affiliations:** 1 Department of General Surgery, Faculty of Medicine, Çukurova University, 01330 Adana, Turkey; 2 Department of Surgical Oncology, Faculty of Medicine, Çukurova University, 01330 Adana, Turkey; 3 General Surgery, Islahiye State Hospital, 27800 Gaziantep, Turkey

**Keywords:** perforated peptic ulcer, older patients, 30-day mortality, blood urea nitrogen-to-albumin ratio, creatinine, prognostic markers

## Abstract

**Objective:** To compare clinical and laboratory markers for 30-day mortality in older patients with perforated peptic ulcer and assess biochemical markers beyond age and American Society of Anesthesiologists (ASA) class. **Methods:** Patients aged ≥65 years undergoing emergency surgery for perforated peptic ulcer from January 2015 to February 2026 were retrospectively analyzed. Discrimination was assessed by the area under the receiver operating characteristic curve (AUC), and incremental value by likelihood-ratio tests and Brier scores. Six marker-specific incremental tests underwent Benjamini–Hochberg false discovery rate (FDR) adjustment. Models incorporating blood urea nitrogen-to-albumin ratio (BAR) or log_2_-transformed creatinine underwent bootstrap internal validation. **Results:** Thirty-day mortality was 37.7% (26/69). Age and ASA class yielded AUCs of 0.785 and 0.755. BAR had the highest univariable biochemical discrimination (AUC, 0.777). Inflammation-based indices showed limited discrimination (AUC, 0.424–0.492); Prognostic Nutritional Index (PNI) had the highest discrimination among immune-nutritional/composite markers (AUC, 0.636; *p* = 0.061). BAR improved the age- and ASA-based model nominally (*p* = 0.023), but not after FDR adjustment (adjusted *p* = 0.068). Creatinine provided the strongest incremental information (AUC, 0.905; Brier score, 0.123; adjusted *p* = 0.008). In the joint model, creatinine improved fit beyond BAR (*p* = 0.024), whereas BAR did not improve fit beyond creatinine (*p* = 0.773). **Conclusions:** Prognostic information was concentrated in age, ASA class, and renal-protein markers. Creatinine provided the most consistent incremental information; BAR showed high univariable discrimination but did not retain significant incremental value after FDR adjustment. Inflammation-based indices remained limited. Independent validation is required.

## 1. Introduction

Perforated peptic ulcer (PPU) remains a surgical emergency associated with high short-term mortality, and the patient population has shifted toward older age groups over time [1]. In population-based data, standardized 30-day mortality after perforated peptic ulcer increased from 8.9% in patients younger than 65 years to 24.2% in those aged 65–79 years and 44.6% in those aged 80 years or older [2]. In a large U.S. surgical cohort of patients aged 65 years or older undergoing open repair for perforated peptic ulcer disease, 30-day mortality increased across successive age groups, from 13% to 18% and 24% [3].

Established approaches to preoperative risk stratification in PPU include the Boey score, Peptic Ulcer Perforation (PULP) score, and American Society of Anesthesiologists (ASA) Physical Status classification [4]. These systems incorporate overlapping clinical risk domains through different definitions and scoring structures [4,5,6]. The Charlson Comorbidity Index (CCI) has also been evaluated as a comorbidity-based prognostic tool in older patients undergoing emergency abdominal surgery [7]. However, the comparative prognostic performance of individual clinical risk components specifically in older patients with PPU has not been the primary focus of these studies [4,5,6,7,8]. The platelet-to-lymphocyte ratio has been evaluated in patients with PPU in relation to mortality and hospital length of stay [9,10]. The Prognostic Nutritional Index has also been investigated as an immunonutritional marker for postoperative morbidity and mortality after PPU surgery [11]. Studies in older patients with PPU have additionally examined other prognostic factors and clinical outcomes, including sarcopenia, while disease-specific scoring research has incorporated selected clinical and biochemical predictors of mortality [8,12,13]. However, these studies have generally examined specific markers, scores, or prognostic domains separately, leaving limited comparative evidence for inflammation- and immunonutrition-based markers in relation to 30-day mortality specifically in older patients with PPU [8,9,10,11,12,13].

Renal and protein-related biochemical variables are incorporated into several PPU-specific prognostic models. The practical scoring system for predicting mortality in patients with perforated peptic ulcer (POMPP) includes age, blood urea nitrogen (BUN), and serum albumin, whereas the more recent simple severity scale for perforated peptic ulcer with generalized peritonitis (the Simple PPUP score) incorporates serum albumin and creatinine among its preoperative predictors [13,14]. Beyond disease-specific scoring systems, the BUN-to-albumin ratio (BAR) has been investigated as a prognostic marker for mortality in older emergency department patients and critically ill surgical patients [15,16]. However, these studies have largely evaluated individual scores or markers in separate populations, leaving limited evidence from direct within-cohort comparisons of clinical score components, renal-protein markers, and inflammation- or immunonutrition-based indices in older patients with PPU [13,14,15,16]. This study aimed to compare the prognostic discrimination of individual clinical and biochemical components represented in established scoring systems with that of complete blood count-, inflammation-, and immunonutrition-based indices for 30-day mortality in older patients with PPU. We further evaluated whether biochemical markers with greater individual discrimination provided incremental prognostic information beyond a clinical model comprising age and ASA class.

## 2. Materials and Methods

### 2.1. Study Design and Population

This retrospective single-center cohort study was conducted at the Department of General Surgery, Çukurova University Faculty of Medicine, between January 2015 and February 2026. Consecutive adult patients who underwent emergency surgery for intraoperatively confirmed gastric or duodenal peptic ulcer perforation were assessed. Patients managed nonoperatively or with insufficient data to determine 30-day mortality or calculate the study variables were excluded.

**Figure 1:**
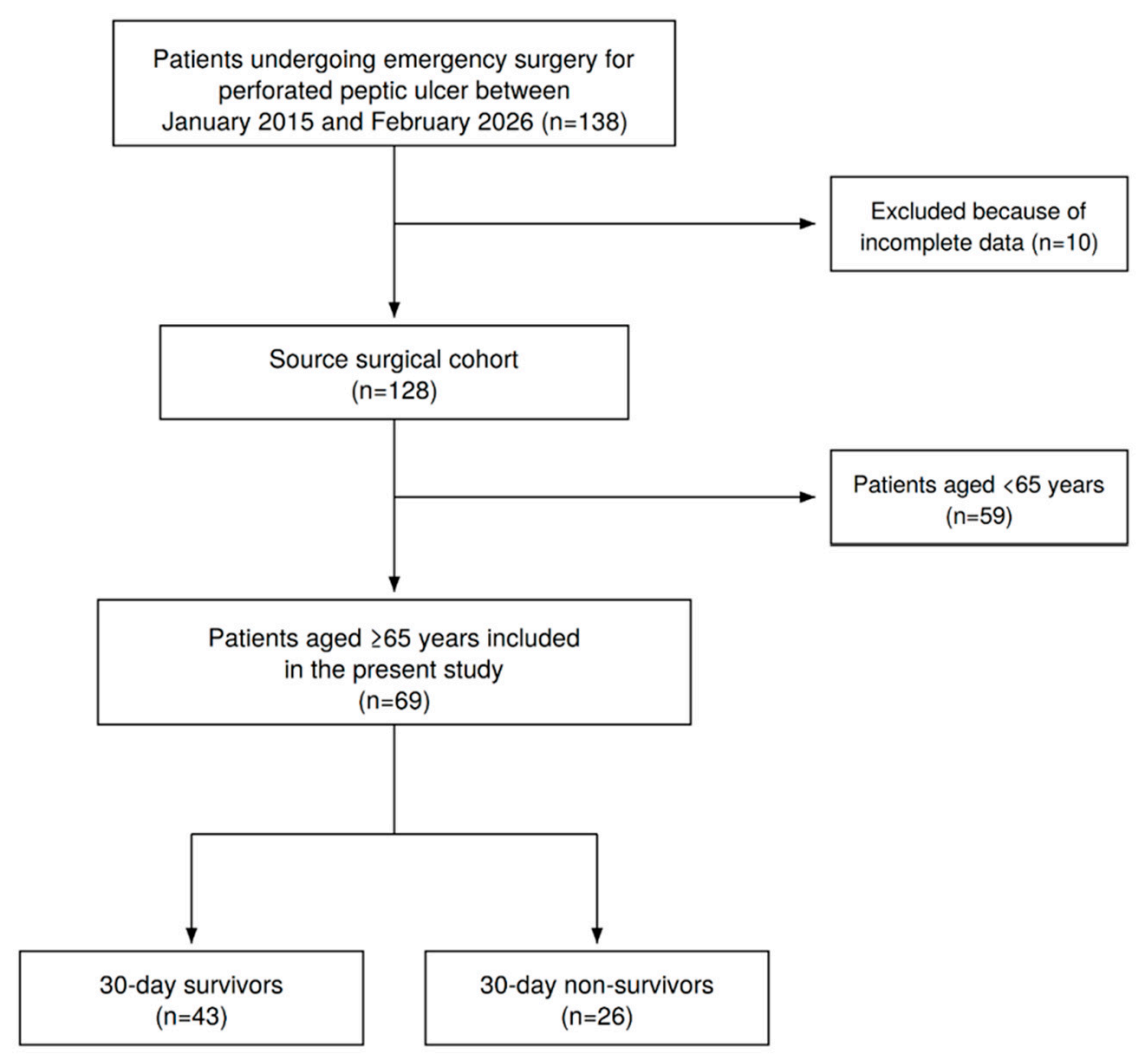
Flow diagram of patient selection for the study.

During the study period, 138 patients were assessed and 10 were excluded because key biochemical data required for the study analyses were incomplete. The source surgical cohort therefore comprised 128 patients, of whom 69 aged ≥65 years constituted the present study population (Figure 1). No imputation of missing values was performed. Because the study included all patients meeting the eligibility criteria during the predefined study period, no a priori sample-size calculation was undertaken. Reporting followed the Strengthening the Reporting of Observational Studies in Epidemiology (STROBE) recommendations.

### 2.2. Data Collection and Definitions

Demographic characteristics, body mass index, comorbidities, American Society of Anesthesiologists (ASA) Physical Status class, nonsteroidal anti-inflammatory drug and steroid use, previous peptic ulcer disease, symptom onset, preoperative vital signs, perforation location, and laboratory measurements were retrospectively obtained from electronic medical records, operative reports, and archived patient files. The first laboratory values available before surgery were used.

The symptom-to-surgery interval was calculated from reported symptom onset to the start of surgery. Delay was additionally categorized as > 24 h. PULP-defined preoperative shock required a systolic blood pressure < 100 mmHg together with a heart rate > 100 beats/min [6]. Prepyloric and other gastric perforations were grouped as gastric or prepyloric locations.

The Charlson Comorbidity Index was calculated without age weighting. For the primary analysis, the peptic ulcer disease component was removed to prevent the index condition from contributing automatically to the score. In a sensitivity analysis, the peptic ulcer disease component was restored while age weighting remained excluded.

### 2.3. Biomarker and Score Calculations

The blood urea nitrogen-to-albumin ratio (BAR) was calculated by dividing blood urea nitrogen in mg/dL by serum albumin in g/dL. The neutrophil-to-lymphocyte ratio (NLR) and platelet-to-lymphocyte ratio (PLR) were calculated from the corresponding absolute cell counts. The systemic immune-inflammation index (SII) was calculated as platelet count × neutrophil count/lymphocyte count.

The Prognostic Nutritional Index (PNI) was calculated as 10 × albumin (g/dL) + 0.005 × absolute lymphocyte count (/µL). The hemoglobin-to-albumin ratio and C-reactive protein-to-albumin ratio (CAR) were calculated by direct division. The hemoglobin-albumin-lymphocyte-platelet index (HALP) was calculated as hemoglobin × albumin × absolute lymphocyte count/platelet count.

The POMPP score assigned one point each for age > 65 years, blood urea nitrogen > 45 mg/dL, and serum albumin ≤ 1.5 g/dL, yielding a total score of 0–3 [13]. The complete PULP score was not included because the primary analyses were designed to compare individual clinical and laboratory components rather than composite prognostic systems. POMPP was retained as a contextual comparator because its components directly represented the age and renal–protein domains evaluated in this study.

### 2.4. Outcome

The primary outcome was all-cause mortality within 30 days after surgery. The 30-day time horizon was selected a priori to evaluate early postoperative mortality and to maintain comparability with established PPU-specific prognostic scoring systems that use 30-day mortality as the principal outcome [6]. Vital status was determined from hospital records and available postoperative follow-up documentation.

### 2.5. Statistical Analysis

Continuous variables are presented as median and interquartile range, and categorical variables as number and percentage. Survivors and non-survivors were compared using two-sided Mann–Whitney U tests for continuous or ordinal variables and Fisher’s exact tests for categorical variables.

Discrimination for 30-day mortality was assessed using receiver operating characteristic (ROC) curves and the area under the curve (AUC). Ninety-five percent confidence intervals (CIs) were estimated using 2000 stratified bootstrap replications, and ROC direction was reversed when lower marker values indicated greater mortality risk. Correlated AUCs were compared using paired DeLong tests for BAR versus POMPP, BAR versus blood urea nitrogen, and the age- and ASA-based models incorporating BAR versus log_2_-transformed creatinine.

BAR, blood urea nitrogen, creatinine, albumin, C-reactive protein (CRP), and the neutrophil-to-lymphocyte ratio were each evaluated for incremental prognostic contribution beyond a reference model containing age as a continuous variable and ASA class as an ordinal variable. Each marker was added separately, and model fit was compared using likelihood-ratio (LR) tests. Performance was summarized using the AUC, Brier score, Akaike information criterion (AIC), and Bayesian information criterion (BIC). The association between BAR and creatinine was assessed using Spearman’s rank correlation coefficient. An exploratory joint model containing age, ASA class, BAR, and log_2_-transformed creatinine was fitted, and nested likelihood-ratio tests evaluated the contribution of each marker beyond the other. Creatinine odds ratios (ORs) represent a doubling in concentration; scaling units for the remaining markers are reported in the tables.

Two additional exploratory reference models were evaluated: age, ASA class, PULP-defined shock, and surgical delay > 24 h; and age with the modified Charlson Comorbidity Index. A BAR threshold of 10.75 was derived post hoc, and its sensitivity, specificity, positive predictive value, and negative predictive value were calculated.

The models incorporating BAR or log_2_-transformed creatinine were internally validated using 2000 bootstrap replications. Optimism-corrected AUCs and calibration slopes were calculated, and apparent calibration was assessed across quartiles of predicted risk. Exploratory decision curve analysis compared the net benefit of the reference model with that of the extended models.

Sensitivity analyses excluded patients with documented chronic kidney disease, assessed the correlation between albumin and C-reactive protein, adjusted the association between albumin and mortality for C-reactive protein, and repeated the comorbidity analyses after restoration of the peptic ulcer disease component. As a sensitivity analysis for multiplicity, *p* values from the six marker-specific incremental tests in the primary age- and ASA-based framework were adjusted using the Benjamini–Hochberg false discovery rate (FDR) procedure. All other exploratory analyses were interpreted using nominal *p* values. All tests were two-sided, and *p* < 0.05 was considered statistically significant. Analyses were performed using Python 3.13.5 with SciPy 1.17.0, statsmodels 0.14.6, and scikit-learn 1.8.0.

### 2.6. Ethical Approval

The study was approved by the Çukurova University Faculty of Medicine Non-Interventional Clinical Research Ethics Committee (approval no. 28; 10 July 2026). The requirement for individual informed consent was waived because of the retrospective design and use of routinely collected clinical data. All data were anonymized, and the study was conducted in accordance with the Declaration of Helsinki.

Artificial intelligence (AI): During the preparation of this manuscript, the authors used ChatGPT (OpenAI) exclusively for English language editing, grammar correction, and minor stylistic refinement to improve readability. ChatGPT was not used to perform statistical analyses or for study design, data collection, interpretation of results, formulation of conclusions, or reference selection. All AI-assisted linguistic outputs were reviewed and edited by the authors, who take full responsibility for the final content of the manuscript.

## 3. Results

### 3.1. Baseline Characteristics of the Study Cohort

A total of 69 patients aged ≥65 years were included, of whom 26 (37.7%) died within 30 days. Perforation was prepyloric in 48 patients (69.6%), gastric at other locations in 5 (7.2%), and duodenal in 16 (23.2%). For the primary analyses, prepyloric and other gastric perforations were grouped together as gastric or prepyloric locations. Non-survivors were older and had higher ASA class and modified Charlson Comorbidity Index scores than survivors. Congestive heart failure was more frequent among non-survivors. Admission blood urea nitrogen, creatinine, and blood urea nitrogen-to-albumin ratio values were higher, whereas albumin concentrations were lower, among patients who died. No significant between-group differences were observed in the symptom-to-surgery interval, PULP-defined preoperative shock, C-reactive protein, or white blood cell count (Table 1).

### 3.2. Discriminative Performance of Clinical Risk Components and Laboratory Markers

Chronological age and ASA class had the largest AUCs among the evaluated clinical variables, at 0.785 and 0.755, respectively. The modified Charlson Comorbidity Index excluding the peptic ulcer disease component had an AUC of 0.648, whereas the AUCs for PULP-defined preoperative shock and a symptom-to-surgery interval > 24 h were 0.484 and 0.502, respectively.

Among the renal-protein markers, the blood urea nitrogen-to-albumin ratio had the largest AUC at 0.777, followed by blood urea nitrogen at 0.750, creatinine at 0.724, and albumin at 0.659. The POMPP score had an AUC of 0.680. The albumin component of POMPP was triggered in only 1 patient (1.4%). POMPP scores were 0 in 2 patients (2.9%), 1 in 47 (68.1%), and 2 in 20 (29.0%); no patient had a score of 3. Thirty-day mortality was 0%, 27.7%, and 65.0% among patients with scores of 0, 1, and 2, respectively. The AUC was higher for BAR than for POMPP (DeLong *p* = 0.030), whereas BAR and BUN did not differ significantly (*p* = 0.348).

The AUCs for C-reactive protein, white blood cell count, neutrophil-to-lymphocyte ratio, platelet-to-lymphocyte ratio, and systemic immune-inflammation index ranged from 0.424 to 0.492. Among the immune-nutritional and composite markers, the Prognostic Nutritional Index had the largest AUC at 0.636 (*p* = 0.061) (Table 2).

### 3.3. Incremental Prognostic Contribution of Laboratory Markers Within the Clinical Reference Framework

**Figure 2:**
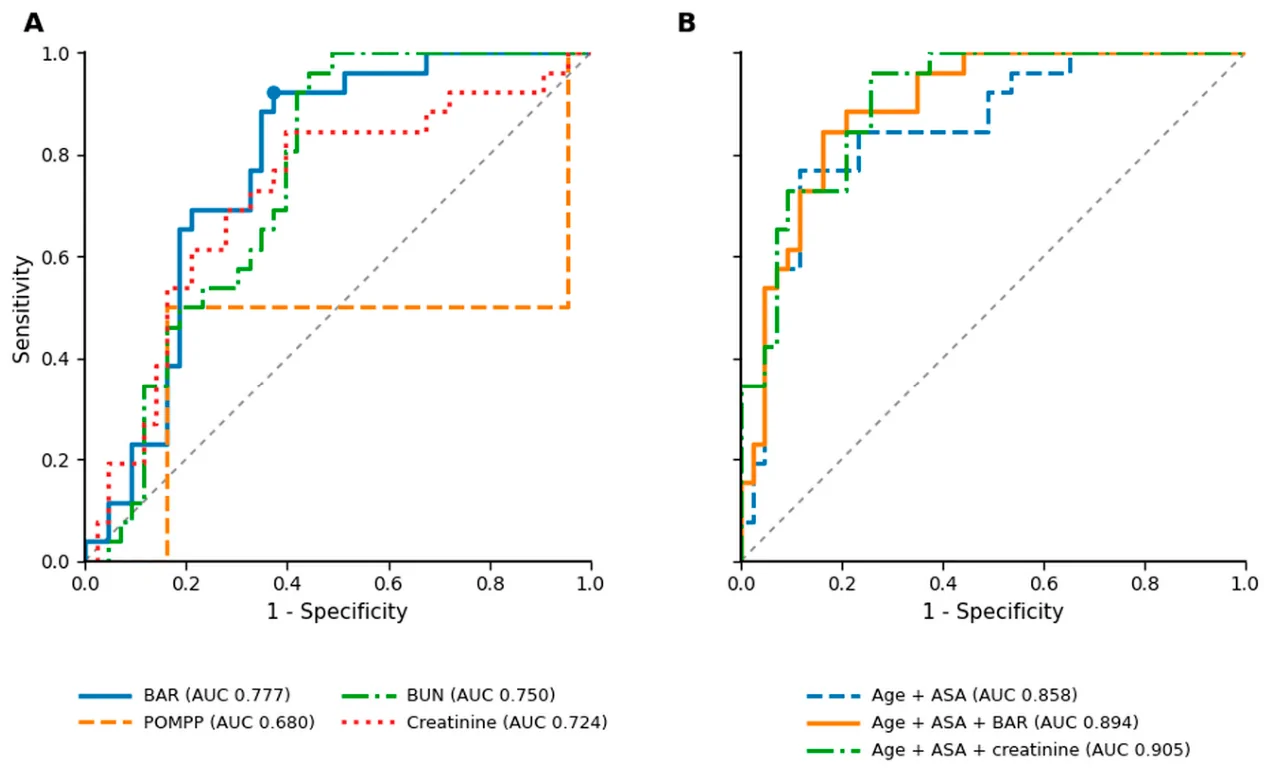
Receiver operating characteristic curves for 30-day mortality. (**A**) Blood urea nitrogen-to-albumin ratio (BAR), blood urea nitrogen (BUN), creatinine, and POMPP score. The point on the BAR curve indicates the post hoc internally derived threshold of 10.75. (**B**) Age- and ASA-based reference model and extended models incorporating BAR or log_2_-transformed creatinine. AUC values are reported in the panel legends. The grey dashed line represents the line of no discrimination. The AUCs of the models incorporating BAR and creatinine did not differ significantly (paired DeLong *p* = 0.538). ASA, American Society of Anesthesiologists; AUC, area under the receiver operating characteristic curve; POMPP, practical scoring system for predicting mortality in patients with perforated peptic ulcer.

The age- and ASA-based reference model had an AUC of 0.858 and a Brier score of 0.140. Addition of BAR increased the AUC to 0.894 and improved model fit (likelihood-ratio χ^2^ = 5.20, *p* = 0.023); each 5-unit increase in BAR was associated with an odds ratio of 1.59 for 30-day mortality (95% CI, 1.04–2.43). Addition of BUN increased the AUC to 0.872 but did not significantly improve model fit (likelihood-ratio χ^2^ = 2.53, *p* = 0.111). Creatinine yielded the largest improvement, increasing the AUC to 0.905 and reducing the Brier score to 0.123 (likelihood-ratio χ^2^ = 10.20, *p* = 0.001); the odds ratio was 4.29 per doubling of creatinine (95% CI, 1.54–11.99). The discrimination of the creatinine model did not differ significantly from that of the BAR model (AUC, 0.905 vs. 0.894; paired DeLong *p* = 0.538). BAR was strongly correlated with serum creatinine (Spearman’s ρ = 0.713, *p* < 0.001). In an exploratory joint model including age, ASA class, BAR, and log_2_-transformed creatinine, discrimination remained unchanged from that of the creatinine model alone (AUC, 0.905), with a Brier score of 0.123, AIC of 60.48, and BIC of 71.65. BAR did not improve model fit beyond creatinine (OR, 1.08 per 5-unit increase; 95% CI, 0.63–1.86; likelihood-ratio χ^2^ = 0.08, *p* = 0.773), whereas creatinine retained incremental value beyond BAR (OR, 3.83 per doubling; 95% CI, 1.07–13.69; likelihood-ratio χ^2^ = 5.08, *p* = 0.024). Addition of albumin increased the AUC to 0.894 and improved model fit (likelihood-ratio χ^2^ = 4.11, *p* = 0.043), although its marker-specific confidence interval included 1.0. Neither C-reactive protein nor the neutrophil-to-lymphocyte ratio improved model fit beyond age and ASA class (*p* = 0.404 and *p* = 0.851, respectively). In the false discovery rate sensitivity analysis, creatinine remained statistically significant (adjusted *p* = 0.008), whereas BAR (adjusted *p* = 0.068) and albumin (adjusted *p* = 0.085) did not retain statistical significance. The corresponding estimates are presented in Table 3 and Figure 2.

### 3.4. Exploratory Analyses in Alternative Clinical Frameworks

In the exploratory framework including age, ASA class, PULP-defined preoperative shock, and a symptom-to-surgery interval > 24 h, the reference model had an AUC of 0.860. Addition of BAR increased the AUC to 0.903 and improved model fit (likelihood-ratio χ^2^ = 5.69, *p* = 0.017). Creatinine increased the AUC to 0.908 and reduced the Brier score to 0.123 (likelihood-ratio χ^2^ = 10.08, *p* = 0.002). Albumin also improved model fit (*p* = 0.029), whereas BUN, C-reactive protein, and the neutrophil-to-lymphocyte ratio did not (*p* = 0.102, *p* = 0.470, and *p* = 0.917, respectively).

In the framework including age and the modified Charlson Comorbidity Index, the reference model had an AUC of 0.792. Creatinine increased the AUC to 0.826 and improved model fit (*p* = 0.007). Addition of BAR increased the AUC to 0.819 without significantly improving model fit (*p* = 0.063). BUN and albumin also did not improve model fit (*p* = 0.165 and *p* = 0.185, respectively) (Table 4).

### 3.5. Exploratory Threshold Analysis for BAR

In a secondary post hoc analysis, a BAR threshold of 10.75 identified 24 of the 26 patients who died within 30 days. Thirty-day mortality was 60.0% among patients with BAR ≥ 10.75 and 6.9% among those with BAR < 10.75. Sensitivity was 92.3%, specificity was 62.8%, positive predictive value was 60.0%, and negative predictive value was 93.1%.

### 3.6. Model Performance and Sensitivity Analyses

**Figure 3:**
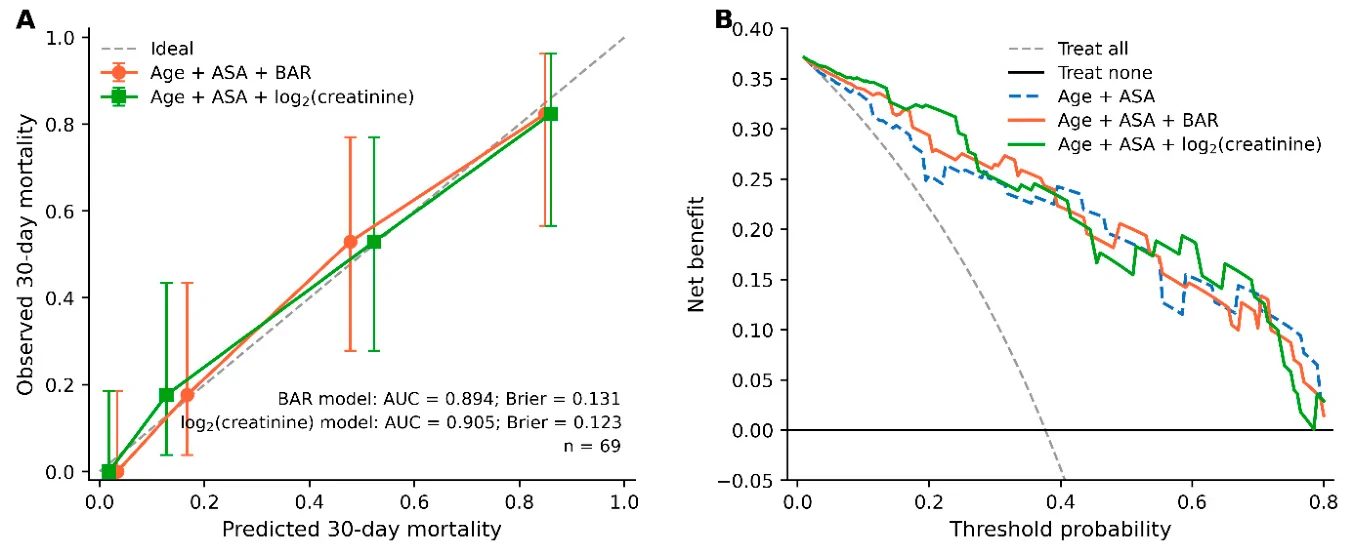
Apparent calibration and decision curve analysis of the models for 30-day mortality. (**A**) Apparent calibration of the age- and ASA-based models incorporating BAR or log_2_-transformed creatinine. Points represent observed 30-day mortality across quartiles of predicted risk, error bars indicate 95% confidence intervals, and the diagonal line represents perfect calibration. (**B**) Exploratory decision curve analysis comparing the age- and ASA-based reference model with the extended models incorporating BAR or log_2_-transformed creatinine. ASA, American Society of Anesthesiologists; AUC, area under the receiver operating characteristic curve; BAR, blood urea nitrogen-to-albumin ratio.

The models incorporating BAR and creatinine had apparent AUCs of 0.894 and 0.905 and Brier scores of 0.131 and 0.123, respectively. After 2000 bootstrap replications, the optimism-corrected AUC was 0.871 for the BAR model and 0.887 for the creatinine model; the corresponding calibration slopes were 0.825 and 0.848. Apparent calibration and exploratory decision curves for the reference, BAR, and creatinine models are shown in Figure 3.

After excluding five patients with chronic kidney disease, 64 patients remained, including 24 deaths. BAR had an AUC of 0.807; when added to the age- and ASA-based model, it increased the AUC from 0.863 to 0.909 and improved model fit (OR, 2.21 per 5-unit increase; 95% CI, 1.18–4.15; LR *p* = 0.006). Creatinine increased the AUC to 0.921 and reduced the Brier score from 0.135 to 0.114 (OR, 6.00 per doubling; 95% CI, 1.69–21.34; LR *p* = 0.001). BAR and creatinine remained strongly correlated (Spearman’s ρ = 0.698, *p* < 0.001); BAR did not improve model fit beyond creatinine (LR *p* = 0.323), whereas creatinine improved model fit beyond BAR (LR *p* = 0.037).

Albumin was not correlated with C-reactive protein (Spearman’s ρ = −0.031, *p* = 0.799). The odds ratio for albumin was 0.42 per 1 g/dL increase before and 0.41 after adjustment for C-reactive protein (*p* = 0.032 and *p* = 0.031, respectively). The non-age-adjusted Charlson Comorbidity Index including the peptic ulcer disease component had an AUC of 0.644 (*p* = 0.037). The corresponding age- and CCI-based model had an AUC of 0.798, which increased to 0.820 with BAR (likelihood-ratio *p* = 0.064) and 0.824 with creatinine (likelihood-ratio *p* = 0.006).

## 4. Discussion

In this study, age and ASA class were the strongest clinical discriminators in older patients with perforated peptic ulcer, whereas renal-protein markers provided greater prognostic information than inflammation-based indices derived from the complete blood count. BAR showed the highest univariable biochemical discrimination, whereas creatinine provided the strongest incremental contribution to the clinical models. In contrast, PULP-defined shock, surgical delay, and inflammation-based markers showed limited performance. These findings suggest that the components of prognostic systems do not carry equal information and that discriminative information is concentrated particularly in age, overall physical status as reflected by ASA class, and variables indicating renal-protein derangement.

The higher discrimination of age and ASA class compared with the modified Charlson Comorbidity Index (mCCI) should be interpreted in the context of the different constructs represented by these measures. Although ASA class is clinician-assigned and therefore partly subjective, it reflects overall physical status and the severity of systemic disease, whereas the CCI quantifies predefined chronic comorbidities using weighted diagnostic categories. Accordingly, the higher AUC observed for ASA in the present cohort may reflect differences in the dimensions of risk captured by the two measures rather than superiority of a subjective assessment over a diagnosis-based comorbidity index. In a study comparing PPU scoring systems, PULP showed the highest discrimination, whereas ASA showed the highest specificity [17]. The prognostic role of the CCI in surgical patients has been reported to be inconsistent in the literature [7]. In our study, excluding age weighting and the peptic ulcer disease component from the mCCI allowed age to be evaluated separately but may also have reduced the discrimination of the index. Although age had the highest AUC among the evaluated clinical variables, this finding should not be interpreted as indicating that age is the single most important or an independent determinant of 30-day mortality. The higher discrimination of BAR compared with POMPP may be related to information loss resulting from categorization of continuous biochemical information using fixed thresholds and to the limited distribution of score components within this older cohort. POMPP assigns points to age, BUN, and albumin using binary thresholds [13]. Categorization of continuous variables has been shown to reduce prognostic performance [18]. In our cohort, the albumin threshold of ≤ 1.5 g/dL was met by only one patient, 68.1% of patients were clustered at a POMPP score of 1, and the restricted age range may have limited the discrimination of the score. This comparison should not be interpreted as evidence of the general superiority of BAR over POMPP, because restriction of the cohort to older patients substantially reduced variability in the age component of POMPP, while its albumin criterion was met by only one patient. Rather, the finding illustrates the limited discrimination of this threshold-based score within the present age-restricted cohort. Although the difference between the AUCs of BAR and BUN did not reach statistical significance, the point estimate favored BAR. An association between BAR and mortality has been reported in older emergency department, critically ill surgical, and geriatric intensive care cohorts [15,16,19]. This parallel suggests that BAR may reflect general renal-protein derangement rather than a PPU-specific process.

In the nominal analysis, adding BAR to the model based on age and ASA class improved model fit; however, this finding did not retain statistical significance after FDR adjustment. In critically ill surgical patients, BAR has been reported to remain associated with mortality after adjustment for demographic, clinical, and laboratory variables [16]. In contrast, although creatinine had lower univariable discrimination than BAR, it showed the strongest and most consistent incremental prognostic contribution in both the nominal analysis and the FDR sensitivity analysis. The strong correlation between BAR and creatinine, together with the lack of improvement in model fit when BAR was added beyond creatinine in the joint model, suggests that the two markers convey largely overlapping prognostic information. The retained incremental contribution of creatinine beyond BAR suggests that the observed overlap may be asymmetric; however, this distinction should be interpreted cautiously given the limited number of outcome events and the resulting imprecision of the joint-model estimates. The biomarker evaluation literature emphasizes that univariable discrimination and the incremental prognostic contribution provided beyond an existing model represent different dimensions of performance [20].

Our creatinine finding is also consistent with recent PPU-specific prognostic literature. In the more recent Simple PPUP score, creatinine and albumin were among the preoperative components selected to predict postoperative adverse events [14]. Because data on dyspnea at rest were unavailable in our cohort, the Simple PPUP score could not be calculated directly. Thorsen et al. found elevated creatinine to be independently associated with 30-day mortality in perforated peptic ulcer [21]. The same study noted that elevated creatinine levels may reflect dehydration, shock, or sepsis in addition to chronic or acute renal dysfunction [21]. Because the analyses in our cohort could not distinguish among these potential processes, creatinine should be interpreted not as a marker of a specific mechanism but as a general prognostic marker reflecting preoperative physiological derangement.

The lack of discrimination of inflammation-based markers in this cohort does not imply that they have no prognostic value in older patients with PPU. Previous PPU studies have associated PLR with mortality and PLR with prolonged hospital stay [9,10]. Among the immune-nutritional and composite markers, PNI showed the highest discrimination, although its association with 30-day mortality did not reach statistical significance (AUC, 0.636; *p* = 0.061). A recent PPU study reported PNI as a potentially useful marker for postoperative morbidity and mortality [11]. However, its discrimination was more limited in our cohort of older patients. The hemoglobin-to-albumin ratio, CAR, and HALP index likewise showed limited discrimination for 30-day mortality. The absence of a marked leukocytosis pattern in our cohort may have contributed to the limited performance of indices derived from blood cell counts. It has been reported that infection in older patients with acute inflammation may not be accompanied by an increase in total or differential leukocyte counts [22]. This may have attenuated the prognostic signal of NLR, PLR, and SII. The influence of capillary leakage and distributional changes on albumin, together with the time-dependent kinetics of CRP, may have limited the prognostic value of CAR derived from a single preoperative measurement [20,23,24]. The limited sample size may also have reduced the ability to detect smaller prognostic contributions.

In our cohort, the high sensitivity and negative predictive value obtained at the BAR threshold of 10.75 suggest that BAR may help identify patients at lower risk. In contrast, its limited specificity and positive predictive value support considering BAR as a marker complementary to clinical risk assessment rather than as a standalone decision-making tool. Although the discrimination of the model incorporating BAR was largely preserved after bootstrap optimism correction, a calibration slope below 1 indicated some degree of overfitting. In the exploratory decision curve analysis, net benefit varied according to threshold probability and model, and no model was consistently superior across the entire threshold range. Therefore, the BAR threshold of 10.75 should be regarded not as a clinical decision threshold but as an exploratory cutoff requiring validation in independent cohorts of older patients with PPU.

The single-center and retrospective design of this study carries the potential for selection bias, misclassification, and unmeasured confounding and limits the generalizability of the findings. Formal frailty measures and validated multidimensional nutritional assessments were not available retrospectively. Although laboratory-derived immune-nutritional markers were evaluated, frailty and broader nutritional status may not have been fully captured, leaving potential for residual confounding in the estimated risk of 30-day mortality. Exclusion of 10 patients with incomplete key biochemical data may have introduced selection bias, although the direction and magnitude of this bias cannot be determined. The limited discrimination of PULP-defined shock and a delay exceeding 24 h should also be interpreted cautiously given the limited number of events and uncertainty in retrospective time records. The presence of only 26 deaths may have reduced the precision of estimates from the multivariable and joint models and increased the risk of overfitting. The four-parameter joint model corresponded to approximately 6.5 outcome events per predictor parameter, further emphasizing the exploratory nature and limited precision of the multivariable estimates. Although bootstrap internal validation evaluates overfitting, it does not eliminate this limitation. Reliance on a single preoperative measurement of biochemical markers precluded assessment of temporal trajectories and may not fully reflect dynamic physiological changes during the acute course of PPU. Consequently, the prognostic value of serial measurements or short-term changes in these markers could not be evaluated. Creatinine measurement cannot distinguish acute renal dysfunction from previously unrecognized chronic renal impairment, and the sensitivity analysis excluding patients with documented chronic kidney disease does not fully resolve this uncertainty. In addition, deriving and evaluating the BAR threshold of 10.75 within the same cohort may have resulted in optimistic estimates of performance. Because the present study was designed specifically around 30-day mortality, the prognostic performance observed here should not be extrapolated to 90-day outcomes. These findings, including the exploratory BAR cutoff, should be externally validated in larger multicenter cohorts representing different clinical settings, with assessment of both discrimination and calibration before clinical implementation.

In this exploratory study, prognostic information in older patients with perforated peptic ulcer was observed to be concentrated particularly in age, ASA class, and renal-protein markers, whereas the discrimination of inflammation-based indices derived from the complete blood count remained limited. BAR and creatinine may be considered candidate markers that stand out across different dimensions of performance; however, these findings should be validated in larger and independent cohorts before translation into clinical use.

## Figures and Tables

**Table 1:** Baseline characteristics of patients aged ≥65 years with perforated peptic ulcer according to 30-day mortality.

Variable	Overall (*n* = 69)	Survivors (*n* = 43)	Non-Survivors *(n* = 26)	*p* Value
Age, years	76 (70–81)	72 (68–79)	80 (77–86)	<0.001
Male sex, *n* (%)	38 (55.1)	27 (62.8)	11 (42.3)	0.135
ASA class	3 (2–3)	2 (2–3)	3 (3–3)	<0.001
Modified Charlson Comorbidity Index excluding peptic ulcer disease	1 (0–3)	0 (0–2)	2 (0–3)	0.031
Congestive heart failure, *n* (%)	11 (15.9)	3 (7.0)	8 (30.8)	0.015
Chronic kidney disease, *n* (%)	5 (7.2)	3 (7.0)	2 (7.7)	1.000
NSAID use, *n* (%)	32 (46.4)	17 (39.5)	15 (57.7)	0.213
Steroid use, *n* (%)	5 (7.2)	3 (7.0)	2 (7.7)	1.000
Previous peptic ulcer disease, *n* (%)	17 (24.6)	8 (18.6)	9 (34.6)	0.158
Symptom-to-surgery interval, h	32 (14–50)	25 (14–45)	32 (16–47)	0.487
PULP-defined preoperative shock, *n* (%)	12 (17.4)	8 (18.6)	4 (15.4)	1.000
Gastric or prepyloric perforation, *n* (%)	53 (76.8)	32 (74.4)	21 (80.8)	0.769
White blood cell count, ×10^3^/µL	8.5 (4.4–12.6)	8.6 (5.3–12.5)	8.2 (3.8–12.7)	0.293
C-reactive protein, mg/L	98.0 (46.6–163.0)	98.0 (56.8–151.5)	97.7 (33.1–235.0)	0.921
Albumin, g/dL	2.87 (2.49–3.30)	2.98 (2.63–3.37)	2.64 (2.03–3.01)	0.028
Blood urea nitrogen, mg/dL	30.9 (20.9–52.2)	22.0 (18.1–36.0)	39.4 (29.3–56.6)	<0.001
Creatinine, mg/dL	1.27 (0.88–2.11)	1.00 (0.78–1.43)	2.01 (1.25–2.23)	0.002
Blood urea nitrogen-to-albumin ratio	12.05 (6.53–17.78)	7.78 (5.39–13.21)	15.24 (12.52–19.69)	<0.001
POMPP score	1 (1–2)	1 (1–1)	1.5 (1–2)	0.002

Data are presented as median (interquartile range) or number (percentage). Continuous and ordinal variables were compared using the Mann–Whitney U test and categorical variables using Fisher’s exact test. PULP-defined preoperative shock was defined as systolic blood pressure < 100 mmHg with heart rate > 100 beats/min. The modified Charlson Comorbidity Index was calculated without age weighting and excluded the peptic ulcer disease component. Gastric or prepyloric perforation included prepyloric and all gastric locations. ASA, American Society of Anesthesiologists Physical Status Classification; NSAID, nonsteroidal anti-inflammatory drug; PULP, Peptic Ulcer Perforation; POMPP, practical scoring system for predicting mortality in patients with perforated peptic ulcer.

**Table 2:** Discriminative performance of clinical risk components and laboratory markers for 30-day mortality.

Marker	AUC	95% CI	Association *p* Value
Clinical risk components			
Age	0.785	0.671–0.882	<0.001
ASA class	0.755	0.639–0.865	<0.001
Modified Charlson Comorbidity Index excluding peptic ulcer disease	0.648	0.521–0.771	0.031
PULP-defined preoperative shock	0.484	0.399–0.581	1.000
Symptom-to-surgery interval > 24 h	0.502	0.382–0.621	1.000
Renal-protein markers			
Blood urea nitrogen-to-albumin ratio	0.777	0.659–0.880	<0.001
Blood urea nitrogen	0.750	0.631–0.859	<0.001
Creatinine	0.724	0.592–0.847	0.002
Albumin	0.659	0.525–0.791	0.028
Disease-specific comparator			
POMPP score	0.680	0.568–0.784	0.002
Inflammation-based markers			
C-reactive protein	0.492	0.343–0.635	0.921
Neutrophil-to-lymphocyte ratio	0.486	0.339–0.628	0.848
Platelet-to-lymphocyte ratio	0.486	0.335–0.642	0.848
Systemic immune-inflammation index	0.431	0.303–0.581	0.343
White blood cell count	0.424	0.283–0.579	0.293
Immune-nutritional and composite markers			
Prognostic Nutritional Index	0.636	0.501–0.769	0.061
Hemoglobin-to-albumin ratio	0.578	0.436–0.725	0.281
C-reactive protein-to-albumin ratio	0.554	0.394–0.695	0.457
Hemoglobin-albumin-lymphocyte-platelet index	0.546	0.387–0.698	0.532

ROC direction was reversed for albumin, the Prognostic Nutritional Index, and the hemoglobin-albumin-lymphocyte-platelet index because lower values of these markers indicated greater mortality risk. Confidence intervals for AUCs were estimated using 2000 stratified bootstrap replications. For continuous and ordinal variables, *p* values were obtained using the Mann–Whitney U test, which is equivalent to testing whether the AUC differs from 0.50. Binary variables were compared using Fisher’s exact test. *p* values in Table 2 are descriptive and were not adjusted for multiple comparisons. PULP-defined shock was defined as systolic blood pressure < 100 mmHg and heart rate > 100 beats/min. ASA, American Society of Anesthesiologists; AUC, area under the receiver operating characteristic curve; CI, confidence interval; PULP, Peptic Ulcer Perforation; ROC, receiver operating characteristic; POMPP, practical scoring system for predicting mortality in patients with perforated peptic ulcer.

**Table 3:** Incremental prognostic contribution of laboratory markers beyond the age- and ASA-based reference model.

Model	Added Marker OR (95% CI)	AUC	Brier Score	LR χ^2^	LR *p* Value	AIC	BIC
Age + ASA	—	0.858	0.140	—	—	66.77	73.47
Age + ASA + BAR	1.59 (1.04–2.43) per 5-unit increase	0.894	0.131	5.20	0.023	63.57	72.50
Age + ASA + BUN	1.29 (0.94–1.76) per 10 mg/dL increase	0.872	0.137	2.53	0.111	66.23	75.17
Age + ASA + log_2_(creatinine)	4.29 (1.54–11.99) per doubling	0.905	0.123	10.20	0.001	58.57	67.50
Age + ASA + albumin	1.67 (0.99–2.82) per 0.5 g/dL decrease	0.894	0.130	4.11	0.043	64.66	73.59
Age + ASA + CRP	1.10 (0.87–1.40) per 50 mg/L increase	0.858	0.139	0.70	0.404	68.07	77.01
Age + ASA + NLR	1.01 (0.94–1.08) per 1-unit increase	0.861	0.140	0.04	0.851	68.73	77.67
Exploratory joint model: age + ASA + BAR + log_2_(creatinine)	BAR: 1.08 (0.63–1.86) per 5-unit increase; creatinine: 3.83 (1.07–13.69) per doubling	0.905	0.123	—	—	60.48	71.65

All models included age and ASA class. Single-marker models were compared with the reference model using likelihood-ratio tests. In the joint model, BAR did not improve model fit beyond creatinine (*p* = 0.773), whereas creatinine improved model fit beyond BAR (*p* = 0.024). AUC and Brier scores are apparent estimates; creatinine was log_2_-transformed. Benjamini–Hochberg false discovery rate-adjusted *p* values for BAR, BUN, creatinine, albumin, CRP, and NLR were 0.068, 0.167, 0.008, 0.085, 0.485, and 0.851, respectively. AIC, Akaike information criterion; ASA, American Society of Anesthesiologists; AUC, area under the receiver operating characteristic curve; BAR, blood urea nitrogen-to-albumin ratio; BIC, Bayesian information criterion; BUN, blood urea nitrogen; CI, confidence interval; CRP, C-reactive protein; LR, likelihood ratio; NLR, neutrophil-to-lymphocyte ratio; OR, odds ratio.

**Table 4:** Exploratory incremental prognostic analyses in alternative clinical frameworks.

Model	Added Marker OR (95% CI)	AUC	Brier Score	LR χ^2^	LR *p* Value	AIC	BIC
Panel A. Established-score-component clinical framework							
Age + ASA + PULP-defined shock + delay > 24 h	—	0.860	0.139	—	—	70.37	81.54
Reference + BAR	1.64 (1.06–2.54) per 5-unit increase	0.903	0.130	5.69	0.017	66.68	80.09
Reference + BUN	1.30 (0.95–1.78) per 10 mg/dL increase	0.878	0.135	2.68	0.102	69.70	83.10
Reference + creatinine	4.27 (1.53–11.93) per doubling	0.908	0.123	10.08	0.002	62.30	75.70
Reference + albumin	1.82 (1.02–3.25) per 0.5 g/dL decrease	0.891	0.129	4.77	0.029	67.60	81.01
Reference + CRP	1.02 (0.97–1.07) per 10 mg/L increase	0.858	0.139	0.52	0.470	71.85	85.26
Reference + NLR	1.00 (0.94–1.08) per 1-unit increase	0.859	0.139	0.01	0.917	72.36	85.77
Panel B. Comorbidity-based clinical framework							
Age + modified Charlson Comorbidity Index	—	0.792	0.178	—	—	79.35	86.05
Reference + BAR	1.40 (0.98–1.99) per 5-unit increase	0.819	0.172	3.46	0.063	77.89	86.83
Reference + BUN	1.22 (0.92–1.62) per 10 mg/dL increase	0.812	0.176	1.92	0.165	79.43	88.36
Reference + creatinine	2.74 (1.26–5.96) per doubling	0.826	0.161	7.37	0.007	73.98	82.92
Reference + albumin	1.34 (0.86–2.09) per 0.5 g/dL decrease	0.819	0.173	1.76	0.185	79.59	88.53

Panel A included age, ASA class, PULP-defined shock, and delay > 24 h; Panel B included age and the modified Charlson Comorbidity Index without age weighting or the peptic ulcer disease component. Each marker was added separately, and likelihood-ratio tests compared extended models with the corresponding reference model. AUC and Brier scores are apparent estimates. AIC, Akaike information criterion; ASA, American Society of Anesthesiologists; AUC, area under the curve; PULP, Peptic Ulcer Perforation; BAR, blood urea nitrogen-to-albumin ratio; BIC, Bayesian information criterion; BUN, blood urea nitrogen; CI, confidence interval; CRP, C-reactive protein; LR, likelihood ratio; NLR, neutrophil-to-lymphocyte ratio; OR, odds ratio.

## Data Availability

The data presented in this study are available on request from the corresponding authors due to privacy and ethical restrictions related to patient records.
